# Machine learning and multi-omics in precision medicine for ME/CFS

**DOI:** 10.1186/s12967-024-05915-z

**Published:** 2025-01-14

**Authors:** Katherine Huang, Brett A. Lidbury, Natalie Thomas, Paul R. Gooley, Christopher W. Armstrong

**Affiliations:** 1 Department of Biochemistry and Pharmacology, Bio21 Molecular Science and Biotechnology Institute, University of Melbourne, Parkville, VIC 3052 Australia; 2 The National Centre for Epidemiology and Population Health, ANU College of Health and Medicine, The Australian National University, Canberra, ACT 2601 Australia

**Keywords:** Precision medicine, Multi-omics, Biomarkers, Machine learning, Artificial intelligence, Data integration, ME/CFS, Heterogeneous illness

## Abstract

Myalgic Encephalomyelitis/Chronic Fatigue Syndrome (ME/CFS) is a complex and multifaceted disorder that defies simplistic characterisation. Traditional approaches to diagnosing and treating ME/CFS have often fallen short due to the condition’s heterogeneity and the lack of validated biomarkers. The growing field of precision medicine offers a promising approach which focuses on the genetic and molecular underpinnings of individual patients. In this review, we explore how machine learning and multi-omics (genomics, transcriptomics, proteomics, and metabolomics) can transform precision medicine in ME/CFS research and healthcare. We provide an overview on machine learning concepts for analysing large-scale biological data, highlight key advancements in multi-omics biomarker discovery, data quality and integration strategies, while reflecting on ME/CFS case study examples. We also highlight several priorities, including the critical need for applying robust computational tools and collaborative data-sharing initiatives in the endeavour to unravel the biological intricacies of ME/CFS.

## Introduction

The chronic illness Myalgic Encephalomyelitis/Chronic Fatigue Syndrome (ME/CFS) evolves and perpetuates from a combination of biological and environmental determinants. Onset often occurs after a trigger event [1], such as viral infection, trauma, or toxin exposure, which induces a physiological response. Such responses are typically transient, however are thought to become persistent or dysfunctional in ME/CFS, manifesting as idiopathic fatigue lasting for 3-months or longer, post-exertional malaise (PEM), brain fog, tender lymph nodes, dizziness, muscle or joint pain, digestive problems, or unrefreshing sleep [2].

The exact biological mechanism that results in a chronic ME/CFS state remains unidentified. However, accumulating evidence indicates anomalies in various biological systems including energy metabolism [3, 4], neuroendocrine function [5], immunology [6, 7], and autonomic regulation [8]. ME/CFS may be considered as a cluster of related, but distinct pathophysiological constructs [9], contrasting the reductionist view of it as a singular entity with stages of disease progression. Additionally, the similarities between long COVID and ME/CFS pathophysiologies [10]—despite long COVID developing from a known viral origin (SARS-CoV-2 infection)—suggest that diverse symptom manifestations may be driven more by individual physiological response, rather than specific underlying causes.

There are currently no definitive laboratory tests, and diagnosis is made based on exclusion. One of the significant challenges in diagnosing ME/CFS is that the symptoms are inherent to a wide range of medical conditions. While comorbid conditions like long COVID, fibromyalgia (FM), and postural orthostatic tachycardia syndrome (POTS) are common in ME/CFS, the more critical diagnostic complication arises from symptom presentations that resemble pre-malignant states, and undiagnosed rheumatic diseases, neurological diseases and endocrinopathies, increasing the risk of misdiagnosis. This not only delays proper treatment but also makes downstream data analysis more difficult by introducing unknown sources of heterogeneity into the patient population. Consequently, the prolonged and unstructured diagnosis and treatment of ME/CFS results in a substantial economic loss, estimated at $14.5 billion in Australia [11] and a minimum of $149 billion in the USA [12], encompassing medical expenses, lost income, disability benefits, and increased use of social services.

The heterogeneous nature and healthcare burden of ME/CFS present a timely opportunity for precision medicine, which aims to understand the molecular and biological factors that initiate and progress human diseases at the individual level [13]. This approach integrates biological data including genetic profiles, medical history, social, and behavioural information, to enable tailored decision-making for disease prevention, prediction, and treatment [14]. For example, in oncology, precision medicine has transformed care through targeted therapies for patients with specific molecular markers, such as HER2 protein in breast cancer [15] and *EGFR* gene mutations in non-small cell lung cancer [16].

However, applying precision medicine to ME/CFS is more challenging due to the lack of well-defined pathology, reproducible biomarkers [17], and identifiable treatment targets. The goal in ME/CFS is to move beyond symptom-based classifications and focus on the biological mechanisms driving the disease [13]. This shift requires advanced computational tools such as machine learning and bioinformatic approaches to model the complex, multi-dimensional data and uncover the key pathways involved in the diverse ME/CFS presentations. Although omics studies have yet to identify definitive pathways in ME/CFS, recent advancements in computational power, growing datasets (data type, volume and sample size), and more efficient machine learning algorithms can reveal previously missed or hidden underlying mechanisms. Once these pathways are identified, the application of precision medicine can be fully realised through endpoints like (differential) biomarker-based diagnostics, patient subgrouping, and personalised treatments targeting specific pathways. This review outlines the essential machine learning steps, key multi-omics findings, and necessary data requirements for future ME/CFS studies implementing these computational frameworks.

### ME/CFS presents new challenges to traditional healthcare

**Fig. 1:**
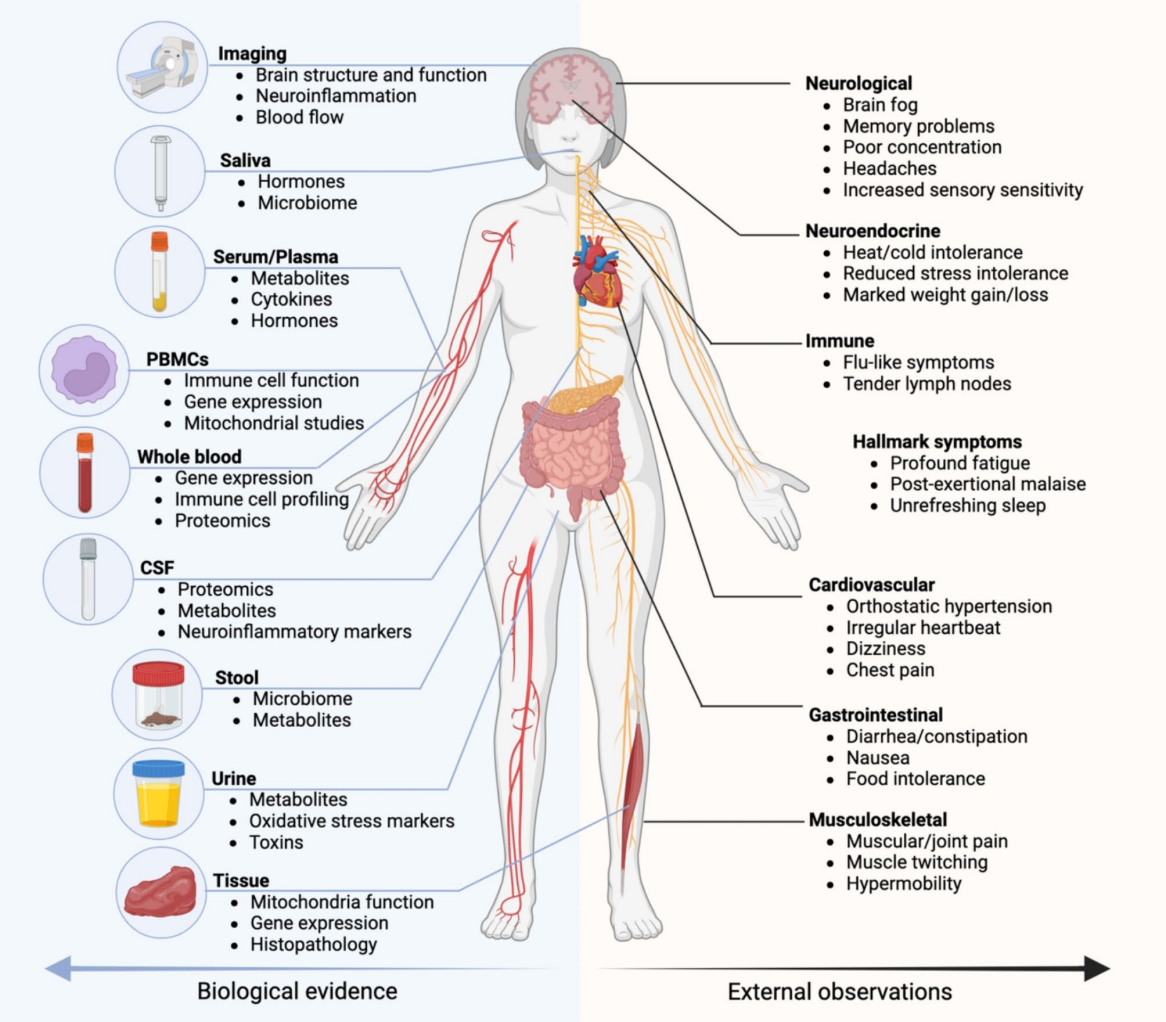
ME/CFS symptom manifestations and biological characterisation opportunities. ME/CFS can be characterised by external symptom observations which affect the neurological, immune, cardiovascular, neuroendocrine, gastrointestinal, and musculoskeletal systems (right). Symptom descriptions are often subjective, however biological characterisation of various biofluids such as saliva, whole blood, and its fractions: serum/plasma, peripheral blood mononuclear cells (PBMCs), cerebral spinal fluid (CSF), stool, urine, and tissue, can offer objective systemic and localised insight into the molecular perturbations that underlie disease pathology and symptoms (left). Relevant analytical experiments including metabolomics, proteomics, microbiome, and biological functions such as blood flow, immune cell function and neuroinflammation are listed below the biofluid types

The traditional procedure for classification of a disease proceeds by identifying the primary dysfunctional organ in which the cardinal symptoms manifest [18]. ME/CFS does not fit neatly into this approach as symptoms can arise from the musculoskeletal, immunological, cardiovascular, gastrointestinal, and neuroendocrine systems (Fig. 1). It challenges the current diagnosis procedure which are based on observable characteristics [19] (generic symptoms and subjective questionnaires) and rely on continuously evolving case definitions (Fukuda 1994 [20], Canadian Consensus Criteria 2003 [21], International Consensus Criteria 2011 [2], National Academy of Medicine 2015 [22], UK National Institute for Health and Care Excellence 2021 [23]). ME/CFS patients undergo extensive family and medical history assessments, series of tests, and may see numerous general practitioners and specialists to receive a clinical diagnosis [24]. Physical examination, clinical measurements and pathology tests often return results within the expected reference range, which does not eliminate ME/CFS diagnosis but can be used to exclude other conditions or to guide further testing.

**Fig. 2:**
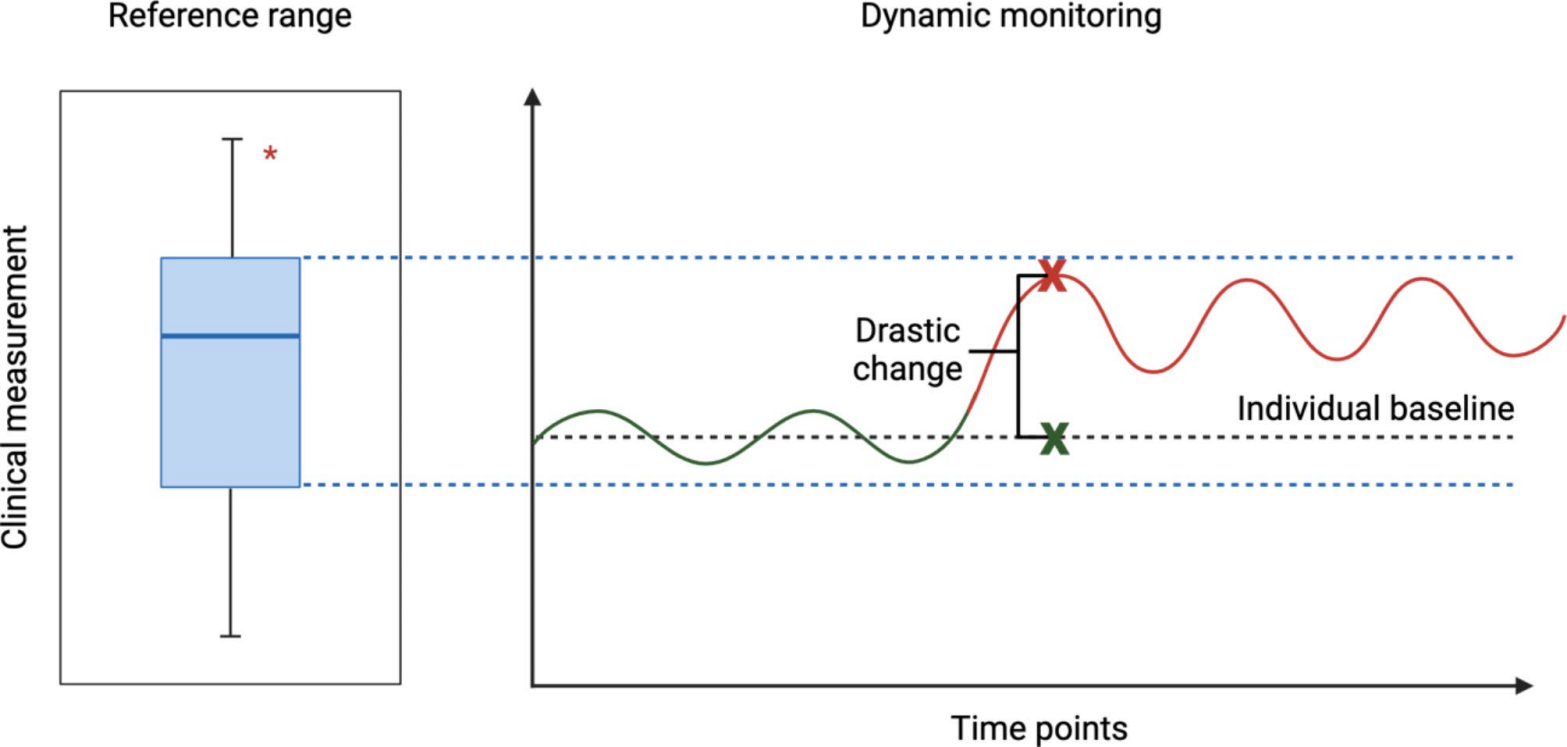
Visual comparison of a conventional reference range and an individual baseline. A conventional reference range is defined by a population average. Here, the reference range is visualised as the interquartile range (blue) of a boxplot (left) with the upper and lower limits extended into the dynamic monitoring panel (right). Biological outliers (*) in the boxplot may lie in the 1st, 4th quartile, or beyond. The dynamic monitoring panel (right) shows an individual baseline (black dashed line) determined by minor fluctuations from continuous monitoring (green solid line). The panel shows a sudden drastic change that may induce an at-risk or afflicted state (red solid line) which does not penetrate the limits of the reference range and eludes detection

The definition of a “reference range” includes lower and upper bounds determined by a population average, with conventional medical practises suggesting only an outlier can indicate an afflicted state. However, this paradigm does not account for the possibility that a shift from an individual’s healthy baseline can occur and still lie within this predefined range [25] (Fig. 2). Patients often have high baseline variation, so what is “normal” for one individual might not be for another. This is relevant for ME/CFS, where symptoms and severity fluctuate, and patients experience periods of relative wellness and exacerbation. Thus, there are limitations to relying on single measurements taken at an isolated timepoint, emphasising the importance of capturing dynamic changes over time in the individual, for both research and clinical settings. Once continuous data is collected, machine learning algorithms such as time series forecasting and anomaly detection, can be employed to model an individual’s baseline, identify deviations from this baseline, and predict adverse events, such as PEM accordingly [26]. Previous small-scale study in intensive care units demonstrated that customised reference ranges significantly reduced false positive alerts [27]. In addition, individualised baselines have been developed to detect COVID-19 pre-symptomatically using wearable data including heart rate and step count [28]. The integration of advanced technologies such as wearable devices (for continuous passive data collection), at-home testing kits (for convenience), and high-throughput profiling now enables repeated, real-time measurements and standardised dynamic data collection, which were not previously accessible or widely utilised.

Treatment strategies for ME/CFS prioritise managing symptoms through social, physical, and occupational support, and energy conservation via pacing [24]. However, similar symptoms can arise from different aetiological events and pathophysiological mechanisms, causing varied responses to the same therapies. Although both pharmacologic and nonpharmacologic interventions are available to alleviate symptoms [29], they are often prescribed on a trial-and-error basis. This uncertainty has led to a growing online community of ME/CFS patients sharing their self-medication experiences, highlighting the urgent need for personalised treatment approaches that target the underlying biology of the individual.

### The promise of “big” biological data

Advances in analytical instrumentation including higher throughput and lower running costs have made deep phenotyping increasingly popular. Such developments have led to comprehensive multi-omics measurements, the collection of imaging data, and detailed physical and pathology results. Here, the term “big data” extends beyond the sheer volume of data to include the hypothesis-generating nature of system biology experiments, where the exploration and analysis of large datasets leads to the formation of a testable hypothesis to initiate a subsequent hypothesis-driven approach [30].

The four main pillars of the omics cascade, genomics, transcriptomics, proteomics, and metabolomics, offer insights into the intricate networks and pathways that drive cellular functions, disease mechanism, and organismal behaviours [31]. Genomics has had the most success in precision medicine, especially in monogenic diseases (due to direct link to a single gene mutation) and cancer applications. The inherent stability of DNA against changes in the environment (cellular or external) facilitates the standardisation of genetic testing in different settings. This contrasts with more sensitive biomolecules like RNA, proteins, and metabolites which require specific analytical instruments or the development of validated assays to be translated into clinical practise. While genomic analyses require advanced bioinformatic methods, once candidate variants are identified, the results can be biologically interpreted in a relatively simple, i.e., binary manner (e.g., presence or absence of a mutation) compared to the continuous and context-dependent variations seen in the downstream omics. Nevertheless, transcriptomics can identify dysregulated gene pathways through gene expression data, while proteomics can validate, or independently identify and quantify proteins and enzymes involved in (mostly downstream) disease processes. Metabolomics offers a dynamic snapshot of the biological state, influenced by genetics, pathogens, diet, lifestyle, and environmental factors, and is valuable for biomarker discovery and real-time tracking of disease progression and treatment response. The various roles of metabolites in multiple pathways (e.g., substrate, intermediate, end-product etc.) can also complicate the linkage of small molecule biomarkers to specific disease mechanisms.

Omics data can be enriched by analysing different biofluids and cell types as they provide complementary insights into the mechanistic roles of potential biomarkers, especially in metabolomics [32]. Each type of biofluid provides either systemic or localised biomarker information. For example, blood serves as a key transporter of circulating nutrients, hormones, and metabolites, reflecting systemic metabolic processes that help maintain homeostasis. Different fractions of blood, such as plasma and peripheral blood mononuclear cells (PBMCs), serve distinct roles—plasma in transport and PBMCs in immune function. Urine captures metabolic by-products and toxins highlighting detoxification pathways and the body’s clearance efficiency. Cerebrospinal fluid (CSF), separated from blood by the blood-brain barrier, mirrors the central nervous system’s biochemical environment, aiding in diagnosing neurological conditions. Saliva contains hormones, antibodies, and proteins useful for non-invasive monitoring of stress, infection, and endocrine function.

While single-biofluid biomarkers may be sufficient as diagnostic biomarkers, the complexity of ME/CFS suggests that correlating biomarker levels in blood with those in other biofluids may offer a more comprehensive understanding of their mechanistic roles. This cross-compartmental correlation is particularly important in ME/CFS, where the interplay between multiple biological systems (e.g., immune, metabolic, and neurological) is likely driving disease pathology. Several biofluids—including interstitial fluid, urine, and sweat—are either directly influenced by blood or result from its filtration and exchange processes. Furthermore, metabolites related to energy metabolism are transported via the bloodstream and consumed at the tissue level. Correlating biomarker levels between these biofluids can validate biological processes by confirming consistent patterns and changes across the different compartments [33]. However, it is important to note that collecting some of these biofluids may require invasive procedures.

Omics technologies are now routinely employed in ME/CFS studies, offering numerous opportunities to explore potential biomarkers. Due to the hypothesis-generating nature of these omics datasets, study outcomes can vary based on several factors including the chosen omics platform, batch effects, sample collection methods, analytical instrument [34], storage and handling. Increasing the sample size is often considered one of the most effective strategies for minimising the influence of technical outliers and strengthening statistical power without indirectly introducing bias. However, this approach can be limited by practical constraints such as cost and data availability. In such cases, normalisation and batch effect correction techniques can be applied post-data acquisition to reduce variability [35], however, different techniques may change study outcomes. When sufficient quality control data or internal standards are available, technical variation can be measured and subsequently removed [36]. Once the data is appropriately pre-processed, advanced bioinformatics tools and machine learning algorithms can be implemented to make sense of multi-modal data and to analyse inter-individual variation.

### Machine learning concepts

State-of-the-art machine learning techniques are increasingly becoming reliable tools for addressing complex biological problems. Machine learning is a branch of artificial intelligence (AI) that aims to emulate human decision making by learning patterns from previous examples drawing on statistics, probability, and optimisation. These patterns are represented as “features” including quantitative, categorical, and unstructured variables such as text or images.

There are three types of machine learning: supervised, unsupervised and reinforcement. Supervised algorithms learn patterns from labelled training data to predict responses, which can be either binary/multi-class (classification) or continuous (regression). Different algorithms can be employed to find patterns without initial data assumptions [37], utilising Boolean logic (AND, OR, NOT), absolute conditionality (IF, THEN, ELSE), conditional probabilities (the probability of X given Y) or optimisation [38], which enable predictions for new input with unknown labels. This method provides more flexibility for data that are non-linear or are interdependent, especially suitable for biological data. Unsupervised learning is employed to identify dissimilarities in unlabelled data for clustering purposes. Reinforcement learning is a dynamic process in which the model trains by reward and punishment mechanisms. Machine learning capabilities that can be applied in ME/CFS, and diseases in general, using classification algorithms involve diagnosis, predicting treatment efficacy and risk susceptibility, and unsupervised algorithms can be employed for disease subtyping via clustering and dimensionality reduction.

When a model is trained on more features than samples (a phenomenon known as the curse of dimensionality), it may become overfitted, meaning that the patterns learnt are too specific to the dataset. Consequently, the model may not make reliable predictions on new input, especially from different data sources. There are various methods to prevent overfitting including feature selection which removes redundant information (see next section), dimensionality reduction and cross-validation. Dimensionality reduction condenses a large number of features into a smaller set that retains the explained variance in the original data. Techniques include principal components analysis (PCA), linear discriminant analysis (LDA) and t-SNE. Cross-validation involves training the model on different subsets of the training data which introduces controlled variability and validates the model on the remaining data subset. Additionally, for a machine learning model to serve as a clinical support tool, it must be interpretable (explainable AI) so clinicians and researchers can understand and trust its predictions. For example, decision trees, regression models, and SHAP (SHapley Additive exPlanations) values [39] provide intellectual oversight by explaining the contribution of individual features to the model’s predictions.

### Classification tasks in ME/CFS

**Fig. 3:**
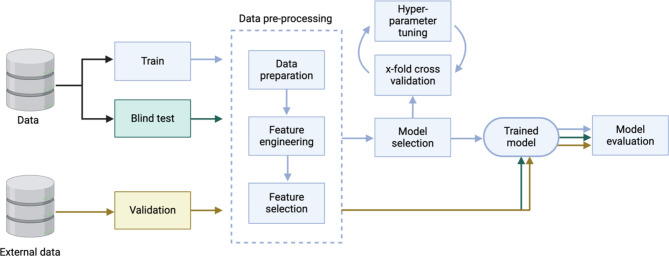
The classification pipeline. The first step in the pipeline involves partitioning the data into training (blue) and blind test (green) sets. Next, the training data is pre-processed with the following steps: data preparation (normalisation and standardisation), feature engineering, and feature selection. Feature engineering is an optional step that transforms raw data into a more informative set of variables. Data pre-processing is performed without exposure to the blind test set. Model selection chooses the optimal machine learning algorithm. During this process, 5- or 10-fold cross validation can be performed with hyper-parameter tuning (e.g., choosing the optimal number of trees in a random forest). The training process is evaluated using cross-validation. Model performance is assessed using the blind test set which is processed using the same parameters as the training set. Finally, a validation set (yellow) from an external data source is required to evaluate the generalisability of the model

The classification pipeline includes data partitioning, data preparation, feature selection, model selection, training, and evaluation with a blind test set (Fig. 3). In ME/CFS, classification applications have focused on biomarker discovery and diagnosis. The main difference between these endpoints is that the biomarker discovery studies typically do not choose a single optimal model; instead, important features from all candidate models are considered as potential biomarkers. Both types of studies are summarised in Table 1, and this section explores the detailed steps involved in these classification applications.

**Table 1:** Summary of recent ME/CFS studies with a machine learning focus

Data	Biofluid	Sample size	Feature selection method (No. features)	Algorithm	Model evaluation	Reference
Metabolomics	Plasma	ME/CFS *n* = 32 HC *n* = 19	ANOVA/F-value (50)	XGBoost	CV only	Yagin et al. (2024) [40]
Raman spectroscopic peaks	PBMCs	ME/CFS *n* = 61 HC *n* = 37	LDA contribution scores (76)	Model stack with GBM	CV, blind test	Xu et al. (2023) [41]
Metagenomics Plasma metabolomics	Stool Plasma	ME/CFS *n* = 154 HC *n* = 79	Top 30 features (top 10 from three individual models)	GBM	CV, external validation	Xiong et al. (2023) [43]
Proteomics and cytokines	Plasma	ME/CFS *n* = 49 HC *n* = 49	Gain (20)	XGBoost	CV only	Giloteaux et al. (2023) [87]
microRNA	Plasma	ME/CFS *n* = 41 ME/CFS + FM *n* = 29 FM *n* = 38 HC *n* = 32	LDA score	Random forest	CV, blind test	Nepotchatykh et al. (2023) [88]
Metabolomics	Plasma	ME/CFS *n* = 26 HC *n* = 26	Impurity score (20)	Random forest	CV only	Yagin et al. (2023) [42]
Antibody, blood pathology	Serum	ME/CFS *n* = 40 HC *n* = 40	Mann-Whitney U test	GBM, XGBoost	CV only	Vogl et al. (2022) [89]
Metabolomics	Plasma	ME/CFS *n* = 106 HC *n* = 91	Bayesian factor	Bayesian model average of five algorithms	CV, blind test	Che et al. (2022) [56]
microRNA	Plasma	ME/CFS *n* = 43 HC *n* = 25	Differentially expressed miRNAs from fold change analysis (11)	Random forest	Blind test only	Neptchatykh et al. (2020) [90]
Routine pathology	Serum	ME/CFS *n* = 85 HC *n* = 17	Impurity score (6)	Random forest, SVM, GBM, decision trees	Training only	Lidbury et al. (2019) [25]
Proteomics	Plasma	ME/CFS *n* = 39 HC *n* = 41	LASSO (20), mean decrease in accuracy (20), gain (20)	LASSO, random forest, XGBoost	CV only	Milivojevic et al. (2019) [91]

ANOVA: analysis of variance, LDA: linear discriminant analysis, GBM: gradient boosted machine, XGBoost: Extreme Gradient Boosting, GLM: generalised linear model, CV: cross validation, SVM: support vector machine

Feature selection simplifies the machine learning model and prevents overfitting caused by high dimensional data (e.g., > 500 features for sample sizes < 100) [38]. There are three main feature selection methods. The first method is a filter approach, which selects features based on statistical tests, independent of the machine learning algorithm. For example, Yagin et al. [40] selected features based on F-value by performing Analysis of Variance (ANOVA) on 892 different plasma metabolomic features. They trained six algorithms on varying decremental feature groups and found that the top 50 features trained on an Extreme Gradient Boosting (XGBoost) classifier was the most optimal model. In another study, Xu et al. [41] performed supervised LDA to extract 76 important features from 1019 Raman spectroscopic peaks based on contribution scores. The advantage of the filter method is the reduced computation burden during model training, though it may not consider the direct contribution of important features to the model’s predictions. The second method is the wrapper method which searches for the best combination of features in the dataset by iteratively adding (forward feature selection) or removing (backward feature selection) features until the best model performance is achieved. This method is algorithm-specific, so different algorithms may choose different feature sets from the same initial features. The third method has feature selection embedded into the algorithm such as LASSO (least absolute shrinkage selector operator) regression. It can involve using algorithm-specific metrics to extract feature importance as demonstrated by Yagin et al. [42]. They trained an initial model on the entire feature set, computed the feature importance using Gini’s impurity score which measures the contribution of each feature to the likelihood of a misclassification, and then retrained the model with the top 20 ranked features.

A blind test set is crucial for evaluating any model or diagnostic tool to ensure unbiased, accurate, and generalisable performance. Best practices involve holding out the blind test set at the start of the machine learning pipeline and not exposing it during training. This step is often overlooked especially if exploratory data analysis and machine learning steps overlap at column-wise data standardisation and filter feature selection. Only a few studies had performed model evaluation using a blind test set, likely due to the limited sample sizes (Table 1). Xiong et al. [43] did not use a blind test set. Instead, they validated their model with data from a second time point (on the same individuals) and an additional external cohort [44]. Their metabolomics-only classifier achieved an area under the receiver operating characteristic curve (AUC) of 0.82 from a 10-fold cross validation, 0.90 for the temporal validation and 0.72 for the external validation. Through temporal validation, they showed that their classifier had stable performance over time, and external validation was possible as raw metabolomics data was shared publicly and acquired using the same analytical platform (Metabolon). In the future, assessing the model with a blind test set should be prioritised to demonstrate a rigorous and unbiased evaluation process.

Model performance can be improved by combining a set of learners into an ensemble model, where the final prediction is derived from aggregating the outputs of several individual models. Ensemble methods such as random forest and XGBoost use decision trees as their base learner but differ in how they aggregate predictions. Random forest employs a technique known as bagging, where multiple decision trees are built on random subsets of the data, and their predictions are averaged, thereby reducing variance and mitigating overfitting. In contrast, XGBoost uses boosting, a sequential method where each new model corrects the errors of the previous one, gradually improving accuracy. Additionally, ensemble models can be constructed through stacking, a method in which different algorithms are combined, with a meta-model learning to optimise the final prediction. For example, Xu et al. [41] used eight different and uncorrelated classification models, each achieving individual accuracies between 47.1 and 61.2%. By stacking these models using a gradient boosted machine model (the meta model) increased the test accuracy to 83.3%.

### Snapshot of ME/CFS omics biomarkers

This section offers an overview of the current state of ME/CFS research into biomarkers (for the main omics levels), highlighting what is known, unknown, needed, and should be prioritised. We do not discuss in detail specific biomarkers as many published reviews have collated findings from genomics [45], immunology [29], and metabolomics [46] studies, and biomarkers in general [17].

The search for genetic markers has focused on identifying single nucleotide polymorphisms (SNPs) through genome wide association studies (GWAS) [47] and candidate gene studies [48, 49]. Traditional GWAS in ME/CFS have lacked statistical power, revealing only a few significant SNPs, which have not been replicated across studies, even within the same UK Biobank cohort. This suggests variation in variant quality control, sample quality control (e.g., ethnicity, gender, relatedness) and analytical methods can lead to discrepancies in the results and require standardised parameters and larger, and more diverse populations. Recently, a combinatorial analysis identified 199 significant SNPs in five biological domains related to viral/bacterial susceptibility, metabolism, autoimmune and sleep. The analysis iteratively tested combinations of 3–5 SNPs in 1000 bootstrapped samples of case and control groups [50]. The sensitive nature of this approach suggested that the identified SNPs were relevant to potential subgroups within ME/CFS, rather than the entire disease. However, the computational burden of fully sampling the entire cohort space and SNP combinations remains a limitation. Nonetheless, this approach targets the polygenicity and heterogeneity of ME/CFS cohorts and represents a shift from seeking a single genetic aetiological variant to recognising multiple common variants [51], each with small effects that can cumulatively contribute to ME/CFS presentations. For future directions these broader searches may be preferable, for example exploring options for generating polygenic risk scores that enhance GWAS association signals [52] and performing a PheWAS [53] on putative ME/CFS SNPs to elucidate genetic signals shared between other diseases or traits.

Transcriptomic studies have examined differentially expressed genes at both the single cell level [54] and the population level [55] in PBMCs before and after an exercise challenge. At the single cell level, ME/CFS patients exhibited increased monocyte dysregulation and was unable to respond to tissue damage caused by high energy demands due to improper platelet activation [54]. At the population level, no significant differences in gene expression of immune cells were found between the two timepoints in ME/CFS, while the healthy control group showed overexpression of genes in multiple pathways [55].

Proteomic, immunologic, and metabolomic studies have demonstrated significant variability, especially when identifying biomarkers in non-invasive and minimally processed biofluids such as serum, plasma, and urine. Not only has a diverse array of biomarkers been identified, but their concentration levels in ME/CFS have also been contradictory. For example, sphingomyelins, a class of lipids involved in cell membrane structure and signalling, were found to be both increased [44], and decreased [56] in ME/CFS patients, suggesting potential disruptions in membrane fluidity or lipid metabolism. Other lipids, such as phosphatidylcholines, ceramides, cholesterol, cholesterol esters, and triglycerides have also generated inconsistent results [4, 44, 56]. This variability highlights the importance of focusing on divergent biochemical pathways rather than individual biomarkers, where both increased and decreased biomarker levels present viable perturbed pathways for different subgroups in ME/CFS. Many studies have identified pathways through pathway enrichment analysis or manual inference from surveying literature. However, a more robust approach would involve validating the findings through integrating multi-omics data, which holistically reflects the state of disrupted pathways in ME/CFS and incorporating longitudinal monitoring.

Understanding whether a biomarker (or a pathway) is merely correlated with ME/CFS or plays a causative role is essential for developing effective treatments [57]. While correlational biomarkers can aid in early detection and monitoring, causal biomarkers can lead to interventions that modify the disease course. Determining causality requires prospective studies, where biomarkers are measured before disease onset and tracked over time. One prospective study identified various dysregulated pathways including glutathione metabolism, nucleotide metabolism, the TCA cycle, glycolysis and urea cycle between individuals that recovered from infectious mononucleosis and those that went on to develop severe ME/CFS [58]. In addition to prospective designs, Mendelian randomisation and randomised controlled trials can further support causal inferences by demonstrating that targeting a biomarker affects disease outcomes [59]. However, in the absence of long-term large scale prospective data for ME/CFS, meta-analysis of case-control studies still offer valuable insights [17, 46, 60]. Meta-analyses can help assess whether reported biomarkers consistently correlate with demographics, symptoms, or external influences, helping to refine biomarker for future causal investigations [61]. While not sufficient for proving causality, this approach is critical for identifying patterns in existing data and guiding the design of future prospective studies.

### Integrating multi-omics and multi-modal data

Multi-omics describes two or more omics, which can be integrated simultaneously or in parallel [62]. The parallel method involves analysing each omics dataset individually and benefits from efficient workflows. Simultaneous analysis considers multiple omics datasets together, offering the advantage of identifying shared sources of variation across different data modalities [63]. This integrated approach contrasts with parallel analysis which does not explicitly link the biological relevance of each individual dataset. Simultaneous integration methods draw on statistical concepts and can be broadly implemented using multivariate analyses, graph-based methods, marginal associations, and unsupervised methods.

The parallel method is analysis-agnostic and offers a simple and flexible solution to multi-omics integration. For example, Xiong et al. [43] integrated three data sets: species abundance (obtained from shotgun metagenomics on gut microbiota), normalised KEGG gene abundance and normalised metabolite profile (plasma metabolomics), into a multi-omics classifier. They first built three individual gradient boosted models for each dataset, ranked features based on their importance and extracted the top 10 features for each model which were then used to train the multi-omics classifier. This approach enabled the identification of potential biomarkers within each omics layer, including low abundance of butyrate-producing microbes and decreased plasma isobutyrate from a correlation analysis post-classification. Kitami et al. [64] performed deep molecular phenotyping on 48 ME/CFS and 52 health controls and collected clinical lab tests, metabolome, immunophenotype, transcriptome and microbiome. They identified 26 significant molecular markers across the five data modalities using two-tailed Mann-Whitney U-test with Benjamini-Hochberg correction and integrated the features using partial least square discriminant analysis.

Multivariate integration methods, applied in simultaneous analysis, are highly effective for disease classification and biomarker elucidation. Giloteaux et al. [65] integrated 353 features including extracellular vesicle (EV) cytokines, plasma cytokines and plasma proteomics with a multi-omics classifier. Feature importance scores were assigned to all the molecular entities based on their direct contribution to the model performance. The top 20 performing features comprised of 15 plasma proteins and 5 EV proteins. This approach contrasts Xiong et al. [43] who had arbitrarily included features into the multi-omics classifier based on prior individual models. Multivariate integrations can also be performed with mixOmics [66] and MetaboAnalyst [66].

Biological networks are complex systems of interconnected components, making graph-based methods ideal for mapping multi-omics interactions [67]. Nagy-Szakal et al. [4] performed a topological data analysis [68] on the AYASDI platform (Ayasdi, Menlo Park, California) integrating 562 plasma metabolites, 574 faecal bacterial relative abundances, 587 metabolic bacterial variables, 61 immune molecules, and 81 questionnaire items for 50 ME/CFS and 50 health controls. To compare how both continuous and categorical variables were related, they used a measure called Jaccard distance, which looks at how dissimilar the variables are between two groups. They also used dimensionality reduction methods to simplify the data for easier visualisation. Their visualisations showed clear class distinctions in the network graphs and showed that bacterial relative abundance features were stronger drivers for class separation than plasma metabolomic features. However, the study lacked biological interpretation from the network analysis, relying instead on univariate logistic regression and independent correlation analysis between bacteria, metabolites, and questionnaire scores (parallel method). Other network analysis approaches include similarity network fusion [69], which is also an unsupervised method that could be used to cluster ME/CFS into subgroups with both discrete and continuous data types.

Marginal association and unsupervised multi-omics integration methods are yet to be rigorously applied in ME/CFS studies but hold significant potential for future applications. Generalised linear models are often employed for case-control studies to identify biomarker associations. Alternatively, marginal association tests can be performed between two different omics, similar to expression quantitative trait loci (eQTLs) analysis, which tests for association of genetic variants and gene expression levels [70], or metabolite GWAS which combines functional genomics and metabolomics [71]. Unsupervised techniques worth exploring include Multi-Omics Factor Analysis (MOFA) [63], and PathME [72], which both have open-source code available for implementation. The primary use case for MOFA is to identify unbroken axes of variation across different data modalities targeted towards heterogenous diseases. Multiple different omics datasets are decomposed into a single matrix comprising of factors $\:\times\:$ samples. These factors can be queried to identify the variance explained by each data modality and the individual contributions of the features using loadings scores. PathME provides direct pathway interpretations and clustering capabilities. Different omics features are first mapped to specific pathways, a score is then assigned to each sample for the different pathways using a sparse denoising autoencoder. Bi-clustering is performed on samples and pathways to generate subgroups.

Here, we have only briefly discussed a few integration methods that could be applied in ME/CFS. As ME/CFS studies are now generating higher volumes of data with greater variety, the application of advanced data integration tools [73] should be prioritised. These tools are more effective and reliable than having researchers manually link significant findings from different datasets, as there is a possibility to miss connections or introduce errors.

### Future endeavours

#### Biobanks

Efforts to build ME/CFS-specific biobanks have gained momentum, with initiatives like the UK ME/CFS Biobank [74], AusME Biobank, and DecodeME project [75] leading the way. The UK ME/CFS Biobank has been instrumental in collecting and providing biological samples and datasets to researchers worldwide. Similarly, the DecodeME project aims to conduct a large-scale genetic study by recruiting 25,000 individuals with ME/CFS, dramatically boosting the statistical power compared to previous studies. In addition, non-disease specific biobanks such as the UK Biobank, Biobank Japan, Estonian Biobank, China Kadoorie Biobank, and the All of Us Research Program in the United States contain vast amounts of genetic, phenotypic, and health data from diverse populations. These resources are invaluable for creating control groups with different ethnicities and for comparing comorbid conditions. Analysing biobanks with linked electronic health records could also help elucidate whether individuals diagnosed with ME/CFS have specific health trajectories that differ from other disease groups [76]. In particular, the ability to cross-reference comorbid diagnoses and sequential disease development provides an opportunity to address the diagnostic ambiguity and develop more precise clinical profiles for ME/CFS. The volume of biobank data also requires standardised collection and processing procedures, ensuring consistency and reliability across timepoints. Consequently, validating multiple small-scale studies with biobank data enhances the accuracy and robustness of their research findings.

#### Data harmonisation and data sharing

There is also the challenge of managing the vast amounts of data collected from small- and medium-scale studies. Studies often employ different questionnaires such as Bell CFIDS disability scale, Chalder fatigue scale, DePaul Symptom Questionnaire, Short Form 36-Item Health Survey, Fatigue Severity Scale, and others, to assess symptoms, severity, and functionality; with each questionnaire having their own focus and format. Developing an intermediary data format that can summarise, or map questionnaire responses to standardised values using schema matching and machine learning [77] would be a more productive solution than continuously creating or updating questionnaires. This approach will facilitate data integration and meta-analysis, allowing researchers to combine and compare results across older and newer studies more effectively. Additionally, the National Institutes of Health (NIH) has also released a data sharing portal, mapMECFS [78], for registered researchers to upload their data, including metadata and biological data, to be compiled into summary statistics. Depositing raw biological data in repositories is also strongly encouraged so different data harmonisation and normalisation strategies can be trialled.

#### Increasing reproducibility

The varying results across ME/CFS omics studies can also be attributed to the different statistical and machine learning methods employed. The exploratory nature of omics studies means researchers often apply various analytical techniques until a novel pattern is detected, which may lead to inconsistent findings. To improve the reproducibility and transparency of these studies, adopting open science practices, such as study pre-registration (e.g., COS Preregistration [79]) and the use of AI/Machine Learning checklists (e.g., AIMe Registry [80]), can be invaluable. Pre-registration ensures that study objectives, hypotheses, and analysis plans are clearly defined in advance, reducing biases and selective reporting. Meanwhile, checklists for AI and machine learning algorithms promote the use of standardised, transparent practises, helping to mitigate the impact of varying analytical approaches. These measures are not meant to restrict research, but to provide a clear distinction between exploratory and confirmatory studies and guide robust hypothesis testing designs in future research.

#### Longitudinal studies

Longitudinal studies provide insights into the progression and fluctuations of ME/CFS. A recent case study (*n* = 1) combined various types of data, such as cytokine profiles and clinical information, with AI techniques like natural language processing and sentiment analysis [7]. This approach extracted functional capacity information from blog posts written during periods of exacerbation, effectively mapping out the patient’s journey through ME/CFS onset, progression, and their responses to different treatments over the span of twenty years. Additionally, the study showcased the untapped potential of integrating electronic health records and personal writings in a retrospective study to identify patterns or signs that could predict disease onset or relapses before physical symptoms appear. Additional tools such as integrative personal omics profile (iPOP) [81] and multiscale, multifactorial response network (MMRN) [82] can provide objective interpretations as sample sizes for longitudinal studies increase.

#### Wearables and digital biomarkers

Integrating digital biomarkers collected through wearable devices offers a transformative approach to monitoring ME/CFS in both longitudinal studies and general settings [83]. Wearables can continuously track various physiological parameters that are highly relevant to ME/CFS. For example, reduced physical activity, temperature, and disrupted sleep patterns can serve as objective indicators of disease severity, while heart rate variability reflects autonomic dysfunction, indicating the body’s stress response and overall cardiovascular health [84]. These insights are unattainable through periodic clinical visits, provide an alternative to patient symptom descriptions, can help establish individualised patient reference ranges and be used to identify early signs of flare-ups. Additionally, because wearable data is passively collected, it mitigates potential sampling bias and captures comprehensive data on both good and bad days. ME/CFS researchers can incorporate continuous digital monitoring into their study designs by utilising platforms like the Digital Medicine Society’s playbook for standard protocols.

#### Embracing AI

While this review primarily focused on machine learning applications, there is also a growing body of research highlighting how deep learning (another branch of AI) can address the heterogeneity of ME/CFS. Deep learning uses neural networks comprising of layers of connected nodes that pass information from one layer to another depending on model parameters such as weights, and biases, and activation functions [85]. The learning process is dynamic and iterative, involving forward propagation to predict output labels and backward propagation (a feedback loop) which adjust the model parameters according to the prediction error, thereby refining the learning process. Deep learning offers several advantages over machine learning for ME/CFS, including the ability to predict multiple outcomes (e.g., phenotypes, clinical scores) for an individual, rather than assigning a single outcome (e.g., ME/CFS or non-ME/CFS label). Hence, this capability is crucial for identifying distinct clinical or biological features of ME/CFS, where heterogeneous individuals can be classified based on their unique combinations of features influenced by symptoms, genetic markers, immune responses, and other biomarkers. Recently, a deep learning framework called BioMapAI was developed to simultaneously integrate microbiome, immune and metabolomic profiles, which were mapped onto 12 clinical symptoms [86]. The model reconstructed clinical symptoms from biological data and elucidated non-linear and biphasic relationships between the two data types through explainable AI [39]. This framework can also be extrapolated to predict other multi-label endpoints, and to include genomic data, demonstrating the effectiveness of deep learning in handling raw, high-dimensional, and multi-modal data necessary to holistically capture the diverse ME/CFS symptomatology.

## Conclusion

There is immense potential for harnessing big data and AI for precision medicine in ME/CFS. The heterogeneity, unestablished aetiology, and suspected multifactorial disease mechanisms in ME/CFS pose significant challenges to biomarker discovery and treatment development, where progress is limited through conventional workflows. Many current studies lack sufficient statistical power, employ diverse study designs, and often rely on manual interpretations of multi-omics data, leading to inconsistent findings. While machine learning and other computational approaches are gaining traction, the limitations of recruitment, small sample sizes, and a lack of standardisation hinder their full potential. However, it is important to acknowledge that AI and machine learning are not magic bullets capable of solving all the complexities of ME/CFS. Their success depends heavily on high-quality, relevant input data and defining specific training endpoints to be modelled. Without these, even the most advanced algorithms may struggle to produce actionable insights. Looking ahead, the future of ME/CFS research looks promising. Continued efforts to expand data integration, biobank resources, transparent reporting, and collaborative research efforts will improve statistical power and reproducibility. With these advancements, we can move from exploratory studies to confirmatory ones, enabling the identification of complex biological patterns at the individual level. Ultimately, these predictive models may positively influence clinical decision making and lead to more effective and personalised treatments for ME/CFS.

## Data Availability

Not applicable.

## Abbreviations

AI: Artificial intelligence
ANOVA: Analysis of variance
AUC: Area under the receiver operator curve
CSF: Cerebrospinal fluid
CV: Cross-validation
eQTLs: Expression quantitative trait loci
EV: Extracellular vesicle
FM: Fibromyalgia
GBM: Gradient boosted machine
GLM: Generalised linear model
GWAS: Genome-wide association study
HC: Healthy control
iPOP: Integrative personal omics profile
LASSO: Least absolute shrinkage selector operator
LDA: Linear discriminant analysis
ME/CFS: Myalgic Encephalomyelitis/Chronic Fatigue Syndrome
MMRN: Multifactorial response network
MOFA: Multi-omics factor analysis
NICE: National Institute for Health and Care Excellence
PBMCs: Peripheral blood mononuclear cells
PCA: Principal component analysis
PEM: Post-exertional malaise
PheWAS: Phenome-wide association study
POTS: Postural Orthostatic Tachycardia Syndrome
SHAP: Shapley Additive exPlanations
SNPs: Single nucleotide polymorphisms
SVM: Support vector machine
XGBoost: Extreme gradient boosting
